# Roles of differential expression of microRNA-21-3p and microRNA-433 in FSH regulation in rat anterior pituitary cells

**DOI:** 10.18632/oncotarget.16615

**Published:** 2017-03-28

**Authors:** Dong-Xu Han, Xu-Lei Sun, Ming-Qiang Xu, Cheng-Zhen Chen, Hao Jiang, Yan Gao, Bao Yuan, Jia-Bao Zhang

**Affiliations:** ^1^ Department of Laboratory Animals, College of Animal Sciences, Jilin University, Changchun, Jilin, P.R. China

**Keywords:** rat anterior pituitary cell, FSH secretion, miR-21-3p, miR-433, animal reproduction

## Abstract

Follicle-stimulating hormone (FSH) secreted by adenohypophyseal cells plays an important role in the regulation of reproduction, but whether microRNAs (miRNAs) regulate the secretion of FSH remains unclear. In the present study, we predicted and screened miRNAs that might act on the follicle-stimulating hormone beta-subunit (FSHb) gene of rats using the TargetScan program and luciferase reporter assays, and the results identified two miRNAs, miR-21-3p and miR-433. We then transfected these miRNAs into rat anterior adenohypophyseal cells and assessed the FSHb expression levels in and FSH secretion by the transfected cells through quantitative PCR and ELISA. The results showed that both miR-21-3p and miR-433 down-regulated the expression levels of FSHb and resulted in the decrease of the secretion of FSH compared with the control group, and treatment with miR-21-3p and miR-433 inhibitors up-regulated the expression levels of FSHb and resulted in the increase of the secretion of FSH. Taken together, our results indicate that miR-21-3p and miR-433 can down-regulate the expression of FSHb by directly targeting the FSHb 3′UTR in rat primary pituitary cells. Our findings provide evidence that miRNAs can regulate FSHb expression and further affect the secretion of FSH and might contribute to the use of miRNAs for the regulation of animal reproduction.

## INTRODUCTION

The pituitary, which is the most important endocrine organ in animals, functions as a main regulator of numerous physiological processes through its control of downstream endocrine glands [1]. Seven types of hormones secreted from the pituitary play important roles in the regulation of organismal activities [2]. Follicle-stimulating hormone (FSH), one of the gonadotropin hormones (GTH), is a pivotal regulator of reproduction [3]. FSH regulates the production of several growth factors that play a vital role in early folliculogenesis and animal reproduction [4–6].

FSH, a glycoprotein hormone encoded by the FSHb gene [7], comprises two subunits, specifically a common α subunit and a unique β subunit, which are involved in specific biological activities [3, 7]. In males, FSH regulates spermatogenesis, and in females, this hormone is indispensable for oogenesis [3]. FSH binds to the follicle stimulating hormone receptor (FSHR) located on Sertoli cells of the testis and granulosa cells of the ovaries to transmit its signal and exert its functions [8]. Numerous studies have indicated that FSH secretion is regulated by many factors. Lower expression levels of gonadotropin releasing hormone receptor (GnRHR) can stimulate maximal production of FSH [9], and at the transcriptional level, FSH secretion is mainly regulated by the gonadotropin releasing hormone (GnRH) signaling pathway [10]. Furthermore, the genetic linkage of novel single-nucleotide polymorphisms (SNPs) within bovine FSHb can influence the serum FSH concentrations [11].

The miRNAs are a family of short, non-coding RNAs that can play crucial roles in both animals and plants [12, 13]. miRNAs can combine with the mRNAs of protein-coding genes to decrease the translational efficiency or modulate the post-transcriptional levels of the mRNAs [14]. Numerous miRNAs have been identified in nearly all metazoan genomes examined since the discovery of the two first miRNAs, lin-4 and let-7 [12, 15, 16], and many studies have reported that miRNAs can influence hormone regulation. For example, miR-26b up-regulates the growth hormone levels by targeting lymphoid enhancer binding factor 1 (Lef-1) in GH3 cells, whereas miR-129-5p, miR-202 and two other miRNAs repress the human growth hormone receptor (GHR) expression levels in both normal and cancer cells [17, 18]. However, it remains unclear whether FSH secretion is regulated by miRNAs.

In this study, we used the TargetScan program, a luciferase reporter assay and quantitative RT-PCR to identify the miRNAs that target the 3′UTR of the FSHb mRNA. In addition, the sequence of the 3′UTR of FSHb mRNA was mutated to validate the interaction between the identified miRNAs and the FSHb mRNA. To investigate whether the identified miRNAs can affect FSH secretion, we transfected the candidate miRNAs into primary rat pituitary cells and measured the FSHb gene expression levels in and the secretion of the FSH hormone from the transfected cells.

## RESULTS

### Identification of miRNAs that potentially target the FSHb 3′UTR

**Figure 1:**
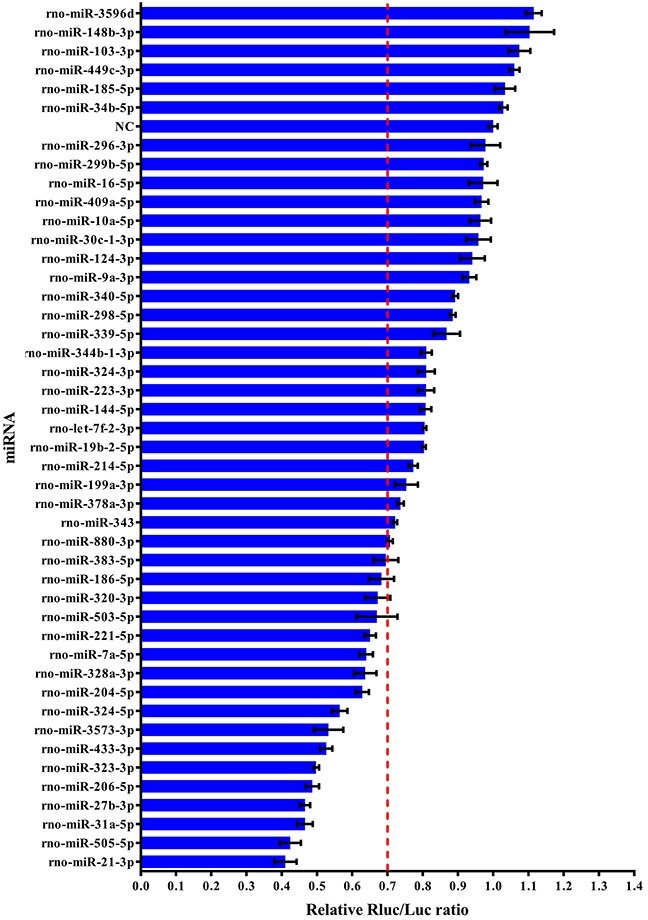
Identification of miRNAs that may target the FSHb 3′UTR Effects of the predicted 45 miRNAs on the reporter gene expression of the pmiR-FSHb-3′UTR-WT vector. Relative luciferase activity was measured 48 h after transfection and normalized to the *Renilla* luciferase activity generated by the co-transfected pmiR-RB-REPORT^TM^ vector. The normalized relative Rluc/Luc activity ratio for the negative control was set to 1.

To identify rat miRNAs that potentially target the 3′UTR of the FSHb gene, we used the TargetScan program to predict 150 miRNAs that could target the 3′UTR of the FSHb mRNA at the post-transcriptional level (Supplementary Table 1). After deleting the duplicate target sites and miRNAs that are not expressed in the rat pituitary based on to the results of a previous microarray study [2], we selected 45 preferred candidate miRNAs (Table 1). To comprehensively verify the interactions between these miRNAs and the FSHb mRNA, we constructed a reporter plasmid, pmiR-FSHb-3′UTR-WT (Supplementary File 2), in which the FSHb 3′UTR was cloned downstream of a firefly luciferase reporter gene. We then used a screening system based on the luciferase reporter plasmid carrying the full-length 3′UTR of the FSHb mRNA and found that 18 miRNAs, specifically miR-433-3p, miR-323-3p, miR-328a-3p, miR-3573-3p, miR-204-5p, miR-206-5p, miR-31a-5p, miR-7a-5p, miR-880-3p, miR-186-5p, miR-503-5p, miR-383-5p, miR-324-5p, miR-505-5p, miR-27b-3p, miR-221-5p, miR-320-3p and miR-21-3p, could suppress the expression of the reporter by more than 30% (Figure 1).

**Table 1:** The final 45 miRNAs

miRNA	Position in the UTR	seed match	context++ score
rno-let-7f-2-3p	926-933	8mer	−0.32
rno-miR-103-3p	761-767	7mer-m8	−0.27
rno-miR-10a-5p	598-604	7mer-m8	none
rno-miR-124-3p	95-101	7mer-1A	−0.01
rno-miR-144-5p	47-53	7mer-m8	−0.02
rno-miR-148b-3p	74-80	7mer-m8	−0.16
rno-miR-16-5p	762-769	8mer	−0.41
rno-miR-185-5p	835-841	7mer-m8	−0.18
rno-miR-186-5p	677-683	7mer-1A	−0.01
rno-miR-199a-3p	1152-1158	7mer-1A	−0.01
rno-miR-19b-2-5p	777-783	7mer-m8	−0.16
rno-miR-204-5p	326-332	7mer-m8	−0.09
rno-miR-206-5p	434-440	7mer-1A	−0.11
rno-miR-206-5p	579-585	7mer-m8	−0.13
rno-miR-21-3p	970-976	7mer-m8	−0.11
rno-miR-214-5p	241-247	7mer-1A	−0.17
rno-miR-223-3p	132-138	7mer-1A	−0.13
rno-miR-27b-3p	714-721	8mer	−0.41
rno-miR-296-3p	308-314	7mer-m8	−0.2
rno-miR-298-5p	71-77	7mer-1A	−0.04
rno-miR-299b-5p	86-93	8mer	−0.27
rno-miR-30c-1-3p	837-843	7mer-m8	−0.18
rno-miR-31a-5p	516-523	8mer	−0.29
rno-miR-320-3p	829-835	7mer-m8	−0.12
rno-miR-323-3p	1368-1374	7mer-m8	none
rno-miR-324-3p	845-851	7mer-1A	−0.07
rno-miR-324-5p	329-336	8mer	−0.55
rno-miR-328a-3p	172-178	7mer-m8	−0.13
rno-miR-339-5p	597-603	7mer-m8	−0.35
rno-miR-340-5p	1219-1225	7mer-1A	none
rno-miR-343	345-351	7mer-m8	−0.25
rno-miR-344b-1-3p	1181-1187	7mer-1A	−0.13
rno-miR-34b-5p	92-98	7mer-m8	−0.29
rno-miR-3573-3p	291-297	7mer-1A	−0.03
rno-miR-3596d	927-933	7mer-1A	none
rno-miR-378a-3p	339-346	8mer	−0.35
rno-miR-383-5p	820-826	7mer-1A	−0.11
rno-miR-409a-5p	639-645	7mer-1A	−0.19
rno-miR-433-3p	1044-1050	7mer-m8	−0.14
rno-miR-449c-3p	674-680	7mer-m8	−0.13
rno-miR-503-5p	763-769	7mer-1A	−0.22
rno-miR-505-5p	111-117	7mer-1A	−0.14
rno-miR-7a-5p	572-578	7mer-1A	−0.01
rno-miR-880-3p	586-592	7mer-1A	−0.11
rno-miR-9a-3p	272-278	7mer-1A	−0.1

### Detection of the expression of the identified miRNAs at different developmental stages

**Figure 2:**
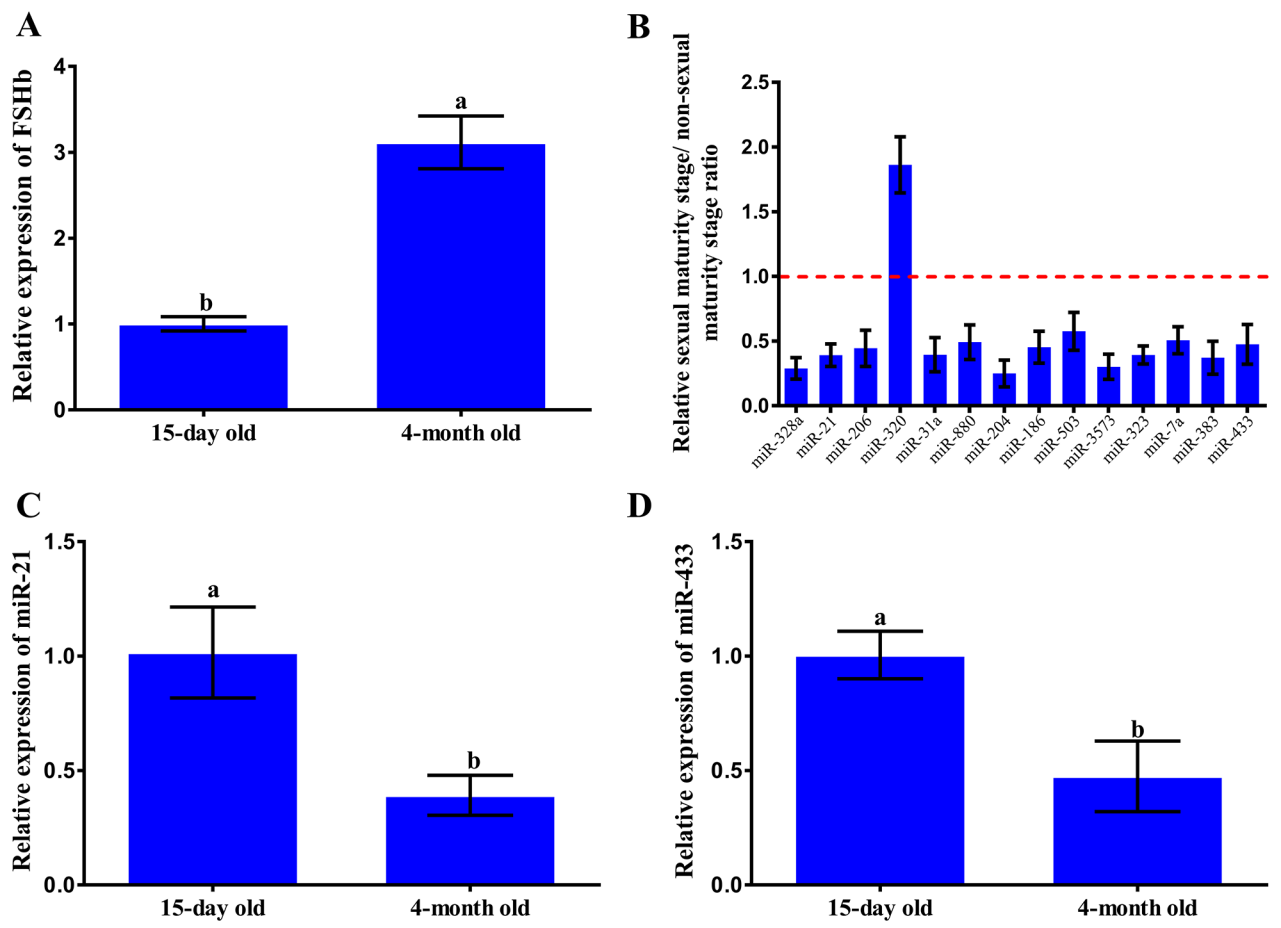
Detection of the expression of the identified miRNAs at different developmental stages **(A)** The expression levels of FSHb were determined via quantitative RT-PCR with GAPDH as an internal standard in the pituitary cells of 15-day-old and 4-month-old rats. **(B)** The expression levels of some of the identified miRNAs were determined through quantitative RT-PCR with U6 as an internal standard at the different developmental stages of the rat pituitary. **(C-D)** In rat anterior pituitary cells from different developmental stages, derived from 15-day-old and 4-month-old rats, the expression levels of miR-21-3p and miR-433 were separately determined via quantitative RT-PCR with U6 as an internal standard. The data are presented as the mean ± standard deviation from at least three independent experiments; statistical significance was determined using multiple comparisons; and P<0.05 was considered significant. The panels with different letters were considered statistically significant (P<0.05).

To determine whether the expression levels of the identified miRNAs varied at different developmental stages, we measured the expression levels of miRNAs that were randomly selected from the set of identified miRNAs at different developmental stages in the rat pituitary. Rats were grouped according to their ages into one of two groups, specifically the 15-day-old and 4-month-old groups, to represent two different developmental stages. To ensure the accuracy of the experiment, we first measured the expression levels of FSHb in the pituitary at the two developmental stages. The results indicated that the level of FSHb was increased (3.12-fold) in the sexual maturity period compared with that detected in the non-sexual maturity period (Figure 2A). Moreover, the results showed that 13 miRNAs were down-regulated in the sexual maturity period compared with their levels in the non-sexual maturity period, whereas one miRNA was up-regulated (Figure 2B). We then randomly selected two miRNAs from the set of 13 down-regulated miRNAs (miR-21-3p and miR-433) for subsequent analysis, and the results suggested that miR-21-3p and miR-433 down-regulated FSHb in the sexual maturity stage compared with the non-sexual maturity stage (Figures 2C and 2D).

### Validation of the interaction between the miRNAs and FSHb *in vitro*

**Figure 3:**
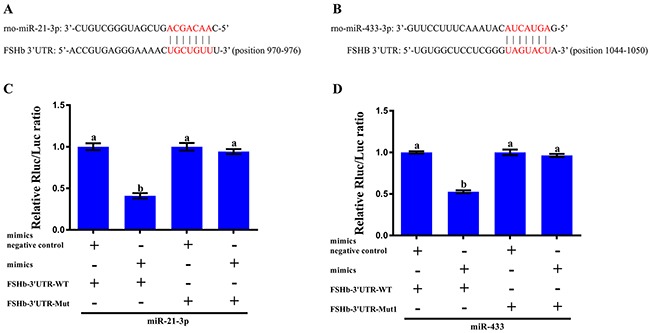
*In vitro* validation of the interaction between the miRNAs and FSHb **(A)** Sequence alignment of miR-21-3p with the 3′UTR of FSHb from the rat. **(B)** Sequence alignment of miR-433 with the 3′UTR of FSHb in the rat. The seed match region predicted by the TargetScan program is indicated in red. **(C)** Relative luciferase activity of the pmiR-FSHb-3′UTR-WT (FSHb-3′UTR-WT) and pmiR-FSHb-3′UTR-MUT (FSHb-3′UTR-MUT) vectors in 293T cells cotransfected with the mimic negative controls and miR-21-3p mimics. **(D)** Relative luciferase activity of the pmiR-FSHb-3′UTR-WT (FSHb-3′UTR-WT) and pmiR-FSHb-3′UTR-MUT1 (FSHb-3′UTR-MUT1) vectors in 293T cells cotransfected with the mimic negative controls and miR-433 mimics. Relative luciferase activity was measured 48 h after transfection and normalized to the Renilla luciferase activity generated through cotransfection with the pmiR-RB-REPORT^TM^ vector. The normalized luciferase activity for the controls was set to 1. The data are presented as the mean ± standard deviation from at least three independent experiments; statistical significance was determined through one-way ANOVA; and P<0.05 was considered significant. The panels with different letters were considered statistically significant (P<0.05).

To examine whether the silencing of FSHb is mediated by the specific, direct interaction of miR-21-3p and miR-433 with the FSHb target site, we predicted the respective target positions of the two miRNAs in the FSHb 3′UTR using the TargetScan program (Figures 3A and 3B), and we then mutated the complementary sites of the two miRNA seed regions to generate pmiR-FSHb-3′UTR-MUT and pmiR-FSHb-3′UTR-MUT1 (Supplementary File 3). We then cotransfected miR-21-3p mimics and pmiR-FSHb-3′UTR-WT into 293T cells, which led to a ~60% reduction in luciferase activity; interestingly, cotransfection of the miR-21-3p mimics and pmiR-FSHb-3′UTR-MUT into 293T cells yielded no significant changes in luciferase activity (Figure 3C). Similarly, co-transfection of miR-433 and pmiR-FSHb-3′UTR-WT into 293T cells yielded a more than a 30% decrease in luciferase activity, whereas cotransfection of miR-433 and pmiR-FSHb-3′UTR-MUT1 yielded no changes in luciferase activity (Figure 3D). Therefore, the results suggested that miR-21-3p and miR-433 can regulate the expression of FSHb.

### Effect of miR-21-3p and miR-433 transfection on rat primary pituitary cells

**Figure 4:**
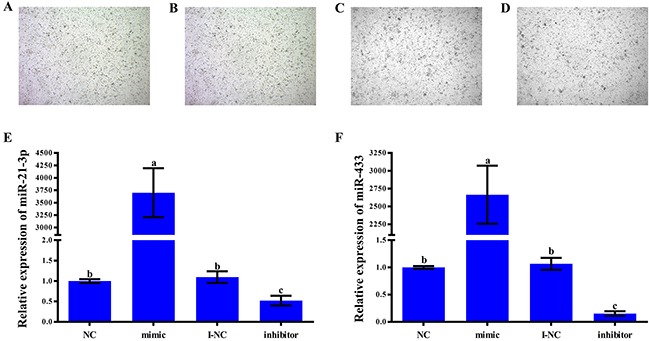
Effect of transfecting rat primary pituitary cells with miR-21-3p and miR-433 After a 24-h transfection period, the rat primary pituitary cells in the blank group **(A)**, the negative control group **(B)**, the miR-21-3p mimic group **(C)**, and the miR-433 mimic group **(D)** are shown. Magnification, 40×. Rat anterior pituitary cells were transfected with the mimic negative controls (NC), mimics, inhibitor negative controls (I-NC) and inhibitors of miR-21-3p and miR-433. **(E-F)** The relative expression of miR-21-3p and miR-433 was detected separately using quantitative RT-PCR with U6 as an internal control. All data are presented as the mean±standard deviation from at least three independent experiments; statistical significance was determined via one-way ANOVA; and P<0.05 was considered significant. The panels with different letters were considered statistically significant (P<0.05).

To detect the growth of cultured primary pituitary cells after transfection with miR-21-3p and miR-433, we observed the growth of the pituitary cells and noted that they appeared to be in good condition (Figures 4A-4D). To determine the transfection efficiency achieved with a 100 nM concentration of the negative controls, mimics, inhibitor negative controls and inhibitors of miR-21-3p and miR-433, we measured the expression levels of the two miRNAs, and the results showed that transfection of the miR-21-3p mimic significantly increased the expression level of miR-21-3p, whereas transfection of the miR-21-3p inhibitor significantly decreased the expression level of miR-21-3p (Figure 4E). We observed a similar pattern for miR-433: the expression level was significantly increased in the mimic group and significantly decreased in the inhibitor group (Figure 4F). These results indicated that the transfection was successful.

### miR-21-3p- and miR-433-mediated regulation of the FSHb expression levels in and FSH secretion by rat primary pituitary cells

To verify that both miR-21-3p and miR-433 target the FSHb gene and to gain further insights into their regulation of reproduction, we measured the expression levels of FSHb in rat primary anterior pituitary cells by quantitative RT-PCR and the secretion of FSH by these cells through ELISA.

**Figure 5:**
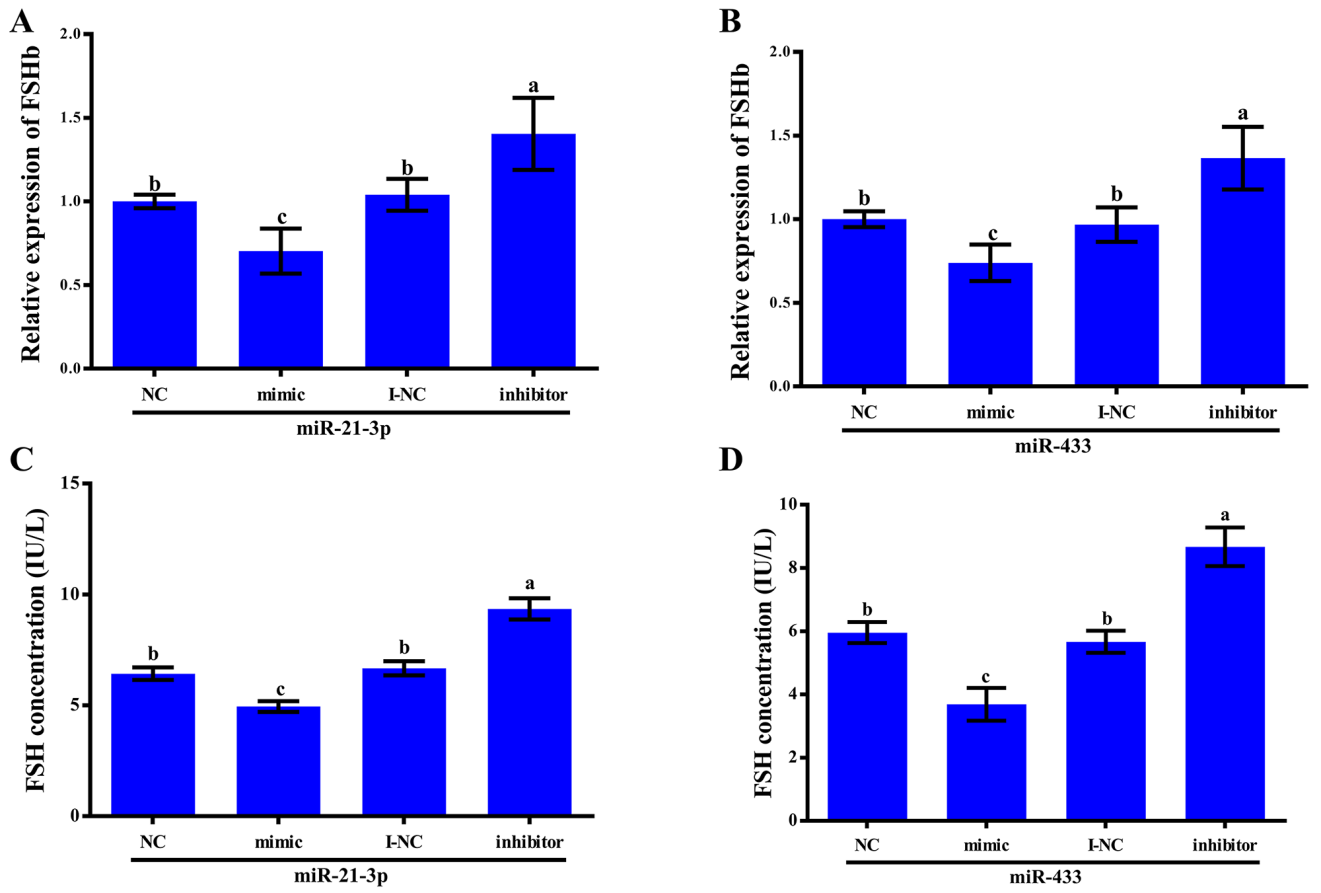
miR-21-3p- and miR-433-mediated regulation of the FSHb expression levels in and FSH secretion by rat primary pituitary cells **(A-B)** Rat anterior pituitary cells were transfected with mimic negative controls (NC), mimics, inhibitor negative controls (I-NC) and inhibitors of miR-21-3p and miR-433. The relative expression of FSHb was detected using quantitative RT-PCR with GAPDH as an internal control. **(C-D)** Rat anterior pituitary cells were transfected with mimic negative controls (NC), mimics, inhibitor negative controls (I-NC) and inhibitors of miR-21-3p and miR-433. A total of 50 μl of supernatant from the pituitary cells was collected and examined with a Rat FSH Elisa Kit at 24 h after transfection. All data are presented as the mean±standard deviation from at least three independent experiments; statistical significance was determined via one-way ANOVA; and P<0.05 was considered significant. The panels with different letters are statistically significant (P<0.05).

We transfected rat primary anterior pituitary cells with the miR-21-3p negative control, mimic, inhibitor negative control or inhibitor at a concentration of 100 nM and subsequently incubated the cells for 24 h. We then measured the expression levels of FSHb in these four groups. Transfection with the miR-21-3p mimic resulted in a 0.70-fold decrease (P<0.05) in the expression level of FSHb, whereas transfection with the inhibitor resulted in a 1.40-fold increase (P<0.05) in the expression level of FSHb (Figure 5A). Moreover, a 0.74-fold decrease (P<0.05) in the FSHb expression level was observed after transfection with the miR-433 mimic, and a 1.37-fold increase (P<0.05) was detected after transfection with the miR-433 inhibitor (Figure 5B). We then measured FSH secretion at the protein level. Interestingly, the results regarding the FSHb expression levels and FSH secretion were similar among the four treatment groups. The FSH concentration was deceased significantly [(6.43±0.28)IU/L vs. (4.95±0.24)IU/L, P<0.05] in the miR-21-3p mimic group compared with the control group and was increased significantly [(6.67±0.32)IU/L vs. (9.35±0.48)IU/L, P<0.05] in the miR-21-3p inhibitor group (Figure 5C). In addition, FSH secretion was decreased [(5.96±0.33)IU/L vs. (3.69±0.52)IU/L, P<0.05] after transfection with the miR-433 mimic and increased after transfection with the miR-433 inhibitor [(5.67±0.35)IU/L vs. (8.67±0.61)IU/L, P<0.05].

These results demonstrated that both miR-21-3p and miR-433 can regulate FSHb expression by directly binding to the FSHb 3′UTR and inhibiting the FSHb expression levels through the degradation of mRNA and inhibition of translation.

## DISCUSSION

miRNAs are post-transcriptional regulators that play significant roles in cancer, disease, growth and development, and stem cell differentiation [19–21]. In addition, miRNAs are expressed in a tissue-specific, time-dependent manner and control the differentiation or maintenance of tissue identity [22]. Furthermore, miRNAs exert effects on the pituitary, including both pituitary adenomas and the normal pituitary. In pituitary adenomas, the miRNA profiles might provide potential markers for predicting the pituitary adenoma histotypes [23]. Previous studies have shown that miRNAs are important emerging elements in many types of adenomas, such as GH-secreting, PRL-secreting, and non-functional pituitary adenomas [24–26]. In addition, miRNAs can affect the development of the normal pituitary. For example, in 2010, Zichao Zhang et al. reported that miR-26b regulates two main factors, Lef-1 and pituitary-specific positive transcription factor 1(Pit-1), during pituitary development [17]. In 2012, Schneeberger et al. reported that changes in miRNA processing structures can lead to pituitary dysfunction and neurodegeneration, which reveals that miRNAs are important in the physiological functions of the pituitary [27]. miRNAs have also been associated with the secretion of hormones in the pituitary, such as adrenocorticotropic hormone (ATCH) and luteinizing hormone (LH) [28, 29]. Because miRNAs are required for the physiological functions and development of the normal pituitary, changes in miRNA expression might cause pituitary disorders. In a previous study, we detected 93 differentially expressed miRNAs and found that seven of these miRNAs were significantly differentially expressed at various developmental stages [2]. Building on these previous results, in the present study, we identified miRNAs that are differentially expressed during the sexual maturity developmental stage compared with the non-sexual maturity stage in the rat pituitary through a microarray analysis, use of the TargetScan program, and a luciferase reporter assay. These differentially expressed mRNAs, which include one up-regulated miRNA and 13 down-regulated miRNAs, represent potential regulators of pituitary development and reproduction and highlight new mechanisms involved in these processes. miR-21-3p and miR-433 were selected for the subsequent experiment.

miR-21 belongs to a microRNA family that is highly expressed in many types of mammalian cells [30]. In previous studies, the expression of miR-21 was associated with human colorectal, breast, and lung cancers as well as human glioblastoma cell lines [31–34]. In 2013, Tushar et al. found that miR-21 targets the SMAD family member 7 (SMAD7) 3′UTR to reduce its expression in hematopoietic cells and plays a role in regulating transforming growth factor-β (TGF-β) signaling [35]. Amitava et al. showed that miR-21 functions in the resolution of wound inflammation [36], and the induction of miR-21 is associated with the silencing of phosphatase and tensin homolog on chromosome ten (PTEN) and programmed cell death 4 (PDCD4), which are tumor-suppressor genes targeted by miR-21 [36–39]. An analysis of pituitary cells revealed that miR-21 is underexpressed (2.4-fold) in ACTH-secreting pituitary adenomas compared with normal pituitary tissues [40]. Moreover, miR-21 is associated with not only pituitary adenomas but also hormone secretion from normal pituitary cells. Our results show that miR-21 can regulate the expression levels of FSHb in rat pituitary cells and reveal that miR-21 might participate in the regulation of animal reproduction in addition to the regulation of pituitary adenomas.

miR-433 is located on chromosome 14q32 and exhibits functions in many types of mammalian cells [41]. Recent reports have shown that miR-433 exerts functions in human tumorigenesis and development [42]. Some researchers have found an association between miR-433 and human glioma, gastric carcinoma, myeloproliferative neoplasms and ovarian, liver and other cancers [42–46]. In another study, Xiao-chun Wang et al. observed that miR-433 acts as a tumor suppressor and inhibits oral squamous cell carcinoma (OSCC) cell growth, invasion and migration by targeting histone deacetylase 6 (HDAC6) [47]. Furthermore, miR-433 inhibits retinoblastoma by suppressing the expression levels of notch homolog 1 (Notch1) and paired box protein Pax-6 (PAX6) [48]. Nevertheless, the role of miR-433 in pituitary adenomas has not been investigated. Regarding hormone secretion, in 2012, Riester et al. reported that ACTH stimulation could modulate the adrenal response by influencing miR-433 to act as an endogenous modulator of the glucocorticoid receptor (Nr3c1) [49]. Similar to miR-21, the role of miR-433 in pituitary hormone secretion remains largely unknown due to a lack of relevant studies. However, our results show that miR-433 can regulate the FSHb expression levels, which provides data on the regulation of hormone secretion in the pituitary by miRNAs.

FSH is an important endocrine hormone that is essential for mammals; therefore, elucidation of the regulatory mechanisms of FSH is particularly noteworthy. Previous studies have indicated that many factors can regulate the expression levels of the FSHb gene or secretion of FSH, such as cis-regulatory elements, relevant hormones, SNPs, and upstream regulators [9, 11, 50]. In 2015, Lisheng Dai et al. reported that a novel cis-regulatory element generated from the 5′-upstream regulatory region of the FSHb gene can influence FSHb expression in bovine cells [51]. In male adult rats, the FSH levels are elevated after pretreatment with a dynorphin (Dyn) antagonist [52]. Other researchers have indicated that a decrease in the serum FSH levels due to the presence of a SNP reduces the induction of FSHb transcription in humans [53]. Although many factors participate in the regulation of FSH secretion, few studies have investigated the miRNAs that regulate FSH secretion. One such study showed that miR-361-3p negatively regulates FSH secretion [10]. Another study found that GnRH exerts an effect on the proliferation of gonadotropic cells [54] and that increasing the number of gonadotropic cells can increase FSH secretion. Moreover, the up-regulation of miR-133 could stimulate GnRH and further impact FSH release [10]. In our study, we found that the up-regulation of miR-21-3p inhibits FSHb expression and leads to decrease of FSH levels, whereas the down-regulation of miR-21-3p results in stimulating FSH secretion. In addition, miR-433 and miR-21-3p exert the same regulatory effects on FSH secretion. Our results provide further evidence of the participation of miRNAs in the regulation of FSH release. Interestingly, we identified 12 other miRNAs (miR-323-3p, miR-328a-3p, miR-3573-3p, miR-204-5p, miR-206-5p, miR-31a-5p, miR-7a-5p, miR-880-3p, miR-186-5p, miR-503-5p, miR-383-5p and miR-320-3p) that might also regulate FSHb expression, and then affected FSH secretion, but their specific effects need to be verified through further experiments.

Taken together, our results show that both miR-21-3p and miR-433 down-regulate FSHb expression and cause FSH secretion decreased. These findings provide insights into the effects of the regulatory mechanisms of miRNAs on the reproductive functions of the pituitary.

## MATERIALS AND METHODS

### Ethics statement

The experiment was strictly conducted in accordance with the guidelines of the Guide for the Care and Use of Laboratory Animals of Jilin University. The protocol was approved by the Institutional Animal Care and Use Committee of Jilin University (Permit Number: 20160312).

### Animals and cell culture

Nine healthy adult male Wistar rats (4 month of age) and four healthy male Wistar rats (15 days of age) were obtained from the School of Medical Science of Jilin University. The four male 4-month old Wistar rats and four male 15-day old Wistar rats were used to detect the expression of the miRNAs at different developmental stages. And other five male 4-month old Wistar rats were for the cell culture and transfection. To improve the cell culture conditions, we covered six-well plates with 10% poly-1-lysine (Boster Biology, China) and placed the plates under a humidified atmosphere containing 5% CO_2_ at 37°C for 12 h. Before using six-well plates, we washed the wells three times with PBS for 5-8 min each at ambient temperature. We euthanized the rats, immediately removed their pituitary glands and placed the glands in ice-cold PBS supplemented with 0.3% BSA (Sigma, USA) and 1% penicillin/streptomycin (HyClone, USA) to wash the blood from the tissue. After washing, the neurohypophysis was removed from the pituitary glands, then the glands were transferred to Dulbecco's Modified Eagle's Medium/Nutrient Mixture F12 (DMEM/F12) (HyClone, USA) containing 2.5% collagenase type I (Gibco, USA), and the glands were then cut into pieces with ophthalmic scissors. We subsequently placed the treated pituitary glands in a water bath at 37°C for 90 min, added PBS to the digested pituitary samples in the wells and placed the mixtures on ice. The cell suspensions were subsequently filtered through a 200-mesh (75-μm) sieve to separate the undigested tissue and cell aggregates and then subjected to centrifugation at 200 g for 10 min. The obtained cell precipitate was diluted with DMEM/F12 (HyClone, USA) containing 15% fetal bovine serum (Gibco, USA), 100 IU/mL penicillin, and 100 μg/mL streptomycin. Finally, 2 ml of the cell suspension was seeded into six-well plates, and the plates were cultured at 37°C under a humidified atmosphere containing 5% CO_2_.

### RNA isolation and quantitative RT-PCR detection

Total RNA was extracted using the miRcute mRNA Extraction and Separation Kit and the TRIzol reagent (Tiangen, Beijing, China) according to the manufacturer's recommended protocol. The total quantity of RNA was assessed with a NanoDrop ND-2000 spectrophotometer (NanoDrop Technologies). We then transformed the total RNA into cDNA using the FastQuant RT Kit (with gDNase) according to the manufacturer's instructions (Tiangen, China). Quantitative RT-PCR was subsequently performed with a Mastercycler ep Realplex^2^ system (Eppendorf, Germany) and SuperReal PreMix Plus (SYBR Green) according to the manufacturer's instructions (Tiangen, China). The mRNA and miRNA primers used in these assays are listed in the Supplementary File 1.

### Transfection of miRNA mimics and inhibitors

All miRNA mimics and inhibitors were purchased from Guangzhou RiboBio Biotech Co., Ltd. The transfection of rat pituitary cells was referred to the previous study [55]. Rat anterior pituitary cells were seeded at a density of 3×10^5^ cells per well in a 24-well plate. Transfection was performed with a Lipofectamine 3000 Transfection Kit (Thermo Fisher Scientific, Waltham, USA) according to the manufacturer's recommended protocol. The final concentrations of the miRNA mimics, inhibitors and negative controls were 100 nM. After transfection, the cells were incubated for 24 h for gene expression analysis.

### Construction of the pmiR-FSHb-3′UTR-WT and pmiR-FSHb-3′UTR-MUT reporter plasmids

The full-length 3′UTR of the rat FSHb mRNA (GenBank Accession No. NM_001007597.2) was cloned between the XhoI and NotI sites of the pmiR-RB-REPORT^TM^ plasmid, generating the pmiR-FSHb-3′UTR-WT plasmid (Supplementary File 2). We introduced site-specific mutations into the pmiR-FSHb-3′UTR-WT plasmid to interrupt the binding sites of miR-21-3p and miR-433, producing the pmiR-FSHb-3′UTR-MUT and pmiR-FSHb-3′UTR-MUT1 plasmids (Supplementary File 3). Guangzhou Ribobio Biotech Co., Ltd., assisted in the construction of the reporter plasmids. All construct products were confirmed via sequencing (Ribobio Biotech Co., Ltd., Guangzhou, China).

### Luciferase reporter assay

To identify the target miRNAs, we cotransfected 293T cells with 45 miRNA mimics, negative controls, pmiR-FSHb-3′UTR-WT and pmiR-RB-REPORT^TM^. For the analysis of miR-21-3p and miR-433, 293T cells were cotransfected with the mimics, negative controls, pmiR-FSHb-3′UTR-WT, pmiR-FSHb-3′UTR-MUT, pmiR-FSHb-3′UTR-MUT1 and pmiR-RB-REPORT^TM^. The 293T cells were seeded at a density of 1.5×10^4^ cells per well with 100 μl of DMEM in 96-well plates and transfected using the Lipofectamine 2000 reagent (Invitrogen, USA). After transfection for 48 h, the luciferase activity was measured using a fluorescence intensity meter (Veritas 9100-002), and the experiments were repeated at least three times. *Renilla* luciferase was used as an internal reference luciferase to minimize experimental variability.

### FSH detection

We collected 50 μl of the supernatant from pituitary cells after incubation for 24 h following transfection. We measured the FSH levels under the different experimental conditions using a Rat FSH ELISA kit according to the manufacturer's instructions (Haling Biotech Co., Ltd., Shanghai, China).

### Statistical analysis

All data are expressed as the means ± standard deviations from three independent experiments. Significant differences were determined via one-way ANOVA for multiple comparisons using SPSS 19.0 for Windows. P<0.05 was considered statistically significant.

## SUPPLEMENTARY TABLES










