# Supplementary material for: Roles of differential expression of microRNA-21-3p and microRNA-433 in FSH regulation in rat anterior pituitary cells
Source: Oncotarget. 2017 Mar 28;8(22):36553–65. doi: 10.18632/oncotarget.16615 (PMC5482676; doi:10.18632/oncotarget.16615)
Supplement: Supplementary file 1 [file oncotarget-08-36553-s001.pdf]

Roles of differential expression of microRNA-21-3p and microRNA-433 in FSH regulation in rat anterior pituitary cells

SUPPLEMENTARY MATERIALS

SUPPLEMENTARY TABLES

Supplementary File 1: Primers used in RT-PCR

See Supplementary File 1

Supplementary File 2: Construction of pmiR-FSHb-3′UTR-WT reporter plasmid

See Supplementary File 2

Supplementary File 3: Construction of pmiR-FSHb-3’UTR-MUT reporter plasmid

See Supplementary File 3

Supplementary Table 1: The 150 miRNAs predicted by TargetScan program

See Supplementary Table 1
