# Supplementary material for: Roles of differential expression of microRNA-21-3p and microRNA-433 in FSH regulation in rat anterior pituitary cells
Source: Oncotarget. 2017 Mar 28;8(22):36553–65. doi: 10.18632/oncotarget.16615 (PMC5482676; doi:10.18632/oncotarget.16615)
Supplement: Supplementary file 2 [file oncotarget-08-36553-s002.docx]

| **primers name** | **sequence(5'-3')** |
| --- | --- |
| U6 RT | CGCTTCACGAATTTGCGTGTCAT |
| miR-21-3p RT | CTCAACTGGTGTCGTGGAGTCGGCAATTCAGTTGAGGACAGCCC |
| miR-433 RT | CTCAACTGGTGTCGTGGAGTCGGCAATTCAGTTGAGACACCGAG |
| U6 F | GCTTCGGCAGCACATATACTAAAAT |
| U6 R | CGCTTCACGAATTTGCGTGTCAT |
| miR-21-3p F | ACACTCCAGCTGGGCAACAGCAGTCGATGG |
| miR-433 F | ACACTCCAGCTGGGATCATGATGGGCTCCT |
| universal reverse | CTCAAGTGTCGTGGAGTCGGCAA |
| GAPDH F | GGAAACCCATCACCATCTTC |
| GAPDH R | GTGGTTCACACCCATCACAA |
| FSHb F | ATACCACTTGGTGTGAGGGC |
| FSHb R | TAGAGGGAGTCTGAGTGGCG |

**S1 File. Primers used in RT-PCR**
