# Supplementary material for: Roles of differential expression of microRNA-21-3p and microRNA-433 in FSH regulation in rat anterior pituitary cells
Source: Oncotarget. 2017 Mar 28;8(22):36553–65. doi: 10.18632/oncotarget.16615 (PMC5482676; doi:10.18632/oncotarget.16615)
Supplement: Supplementary file 3 [file oncotarget-08-36553-s003.docx]

**S2 File. Construction of pmiR-FSHb-3’UTR-WT reporter plasmid**

The full-length 3’UTR of rat FSHb mRNA (NM_001007597.2) was cloned between the XhoI and NotI sites in the pmiR-RB-REPORT^TM^ plasmid, forming the pmiR-FSHb-3’UTR-WT plasmid. The length of the product was 1199 bp. The primers used for PCR were as follows:

FSHb-3’UTR F: GCGCTCGAGGGAACAATGGACATTGCC

FSHb-3’UTR R: AATGCGGCCGCTTCATCAGTAGCACTTTTA

The PCR product was analyzed via agarose gel electrophoresis. Its length was

1199 bp, matching the theoretical length (Fig. 1A). The PCR colony was identified after purification of the PCR product, enzyme cleavage, purification of the cleavage product, connection and convention. The length of the product from the colony was 1500 bp, similar to the theoretical length (Fig. 1B).


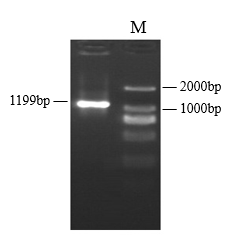

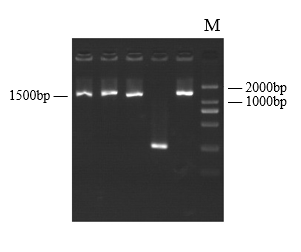


A B

**S1. Fig. 1. Results of agarose gel electrophoresis.** (A) The length of the PCR product was 1199 bp according to the markers. (B) The length of the product from the colony was 1500 bp according to the markers.

The results of sequencing the positive clone were as follows (the underlined sequence is the inserted sequence, and the sequence on both sides is the vector sequence):

GGACGCTCCAGATGAATGGGTAAGTACATCAAGAGCTTCGTGGAGCGCGTGCTGAAGAACGAGCAGTAATTCTAGGCGATCGCTCGAGGGAACAATGGACATTGCCATTCACCCACCCTTGTCTTGAAGGACCATGATATCCAAAATGTCTGGGTCTGCTCTGCACTGGGGCTGTAAACCACTGCCTTAGGGACCATCGGCTCCACTATTGTCCACTGTACTGACAGGTAGTGAGGAGGAGCTCAGGACTGGGAGTGCCAGGGCCAGGACTCTATACCACTCATCTTTCCCTCCATCCACACTGTTGATTATCTAAGTCATTCACTCTCAACTCACAGCTTGGGGCGGGGGGGTTGGAGGCTTTAATTTTCAGCAATCTTAGGAATCTTCTAGAGCAACCCTTTCCTTTAGACAAAGGGATGCATGAGTCCAGAGGGAGGAAAGGAAAGTGGAAATCTGTGAAAGAACTAAACCTAGCATAACCATCTGCTTTTCAGAGCCATCAGACTTTTAAAGACTCCAGCATGATTGCAAGCGAAAGGTCTAAAGTCTGCATGGAATTCAATGAGAAACTCAATGGTTTCCTTAAGGAAAAGAAAGGCAAGTCTTGCCACATATGCCAGGAAATGCTGACGTAGCTGGCTGAGGAAAGCTGTCCTCCACAAGGTTAGGATACTCAACTCTTCCAAAGCATGTGGAGTATTGAGACAGGGTTGAACATTTGTGGATTGTTCTATGCAGATGAAGACAGAGGAAGTAACCAAGCTCAGAATTTCCAAGGTATTGCCACCAAGTTCTTTAGTTAAAGGAAAAGAAAAAAATTAATTTGGAACTGTGAATATAGTCTACATTTATTCATTACTTTAAGAAATGATCAAATGCTGCTAGGATTCTGCAAAACTCCCTTGGAATCAAAGAAAACAACAGTGAAAGTGTTTGATAGGTGATCTAACCAGCTTTCTCTCCCATGCAGTGAGAGAAGGGTTTGAAGTGGGTTTCAAAGAAGTTTCAGTGATTAATTAAATACATTTAACTTGCCTACAAATACACCTGTATAAATTAATGGTTATATAAAATTAACCGTGAGGGAAAACTGCTGTTTTCTAGGACCCCCTGCTTCTCTTTTACCTATCCACATTCTCCTTAATCTTTTGTTCCTTTCAAATACATCATGAGTCAAATTCTTTTAAAGCTGTTAACTCTTTTCATCATGTACCTATTTCACAGTTATGATGTTTGATTAATTTATTTAAATCTTATTTTTTTAATAAAAGTGCTACTGATGAAGCGGCCGCTGGCCGCAATAAAATATCTTTATTTTCATTACATCTGTGTG

The length of the rat FSHb gene was 1655 bp, and the full sequence (NM_001007597.2) was as follows:

GTTCAGCTTTCCCCAGGAGAGATAGCCAACTGCACAGGACATAGCTGTTGACTTACCTGGCCATGATGAAGTCGATCCAGCTTTGCATCCTACTCTGGTGCTTGAGAGCAGTCTGCTGCCATAGCTGTGAACTGACCAACATCACCATCTCAGTAGAGAAGGAAGAGTGCCGTTTCTGCATAAGCATCAATACCACTTGGTGTGAGGGCTACTGCTACACCAGGGATCTGGTGTATAAGGACCCAGCTAGACCAAACACCCAGAAAGTATGCACCTTCAAGGAGCTGGTGTACGAGACCATAAGATTGCCTGGCTGTGCCCGCCACTCAGACTCCCTCTACACATATCCAGTAGCCACTGAATGCCACTGTGGCAAGTGTGATAGTGACAGCACCGACTGCACTGTAAGAGGCCTGGGACCCAGCTACTGCTCCTTCGGTGAAATGAAAGAATAAGGAACAATGGACATTGCCATTCACCCACCCTTGTCTTGAAGGACCATGATATCCAAAATGTCTGGGTCTGCTCTGCACTGGGGCTGTAAACCACTGCCTTAGGGACCATCGGCTCCACTATTGTCCACTGTACTGACAGGTAGTGAGGAGGAGCTCAGGACTGGGAGTGCCAGGGCCAGGACTCTATACCACTCATCTTTCCCTCCATCCACACTGTTGATTATCTAAGTCATTCACTCTCAACTCACAGCTTGGGGCGGGGGGGTTGGAGGCTTTAATTTTCAGCAATCTTAGGAATCTTCTAGAGCAACCCTTTCCTTTAGACAAAGGGATGCATGAGTCCAGAGGGAGGAAAGGAAAGTGGAAATCTGTGAAAGAACTAAACCTAGCATAACCATCTGCTTTTCAGAGCCATCAGACTTTTAAAGACTCCAGCATGATTGCAAGCGAAAGGTCTAAAGTCTGCATGGAATTCAATGAGAAACTCAATGGTTTCCTTAAGGAAAAGAAAGGCAAGTCTTGCCACATATGCCAGGAAATGCTGACGTAGCTGGCTGAGGAAAGCTGTCCTCCACAAGGTTAGGATACTCAACTCTTCCAAAGCATGTGGAGTATTGAGACAGGGTTGAACATTTGTGGATTGTTCTATGCAGATGAAGACAGAGGAAGTAACCAAGCTCAGAATTTCCAAGGTATTGCCACCAAGTTCTTTAGTTAAAGGAAAAGAAAAAAATTAATTTGGAACTGTGAATATAGTCTACATTTATTCATTACTTTAAGAAATGATCAAATGCTGCTAGGATTCTGCAAAACTCCCTTGGAATCAAAGAAAACAACAGTGAAAGTGTTTGATAGGTGATCTAACCAGCTTTCTCTCCCATGCAGTGAGAGAAGGGTTTGAAGTGGGTTTCAAAGAAGTTTCAGTGATTAATTAAATACATTTAACTTGCCTACAAATACACCTGTATAAATTAATGGTTATATAAAATTAACCGTGAGGGAAAACTGCTGTTTTCTAGGACCCCCTGCTTCTCTTTTACCTATCCACATTCTCCTTAATCTTTTGTTCCTTTCAAATACATCATGAGTCAAATTCTTTTAAAGCTGTTAACTCTTTTCATCATGTACCTATTTCACAGTTATGATGTTTGATTAATTTATTTAAATCTTATTTTTTTAATAAAAGTGCTACTGATGAAA

When the two sequences were aligned in NCBI, 100% identity was observed (Fig. 2).


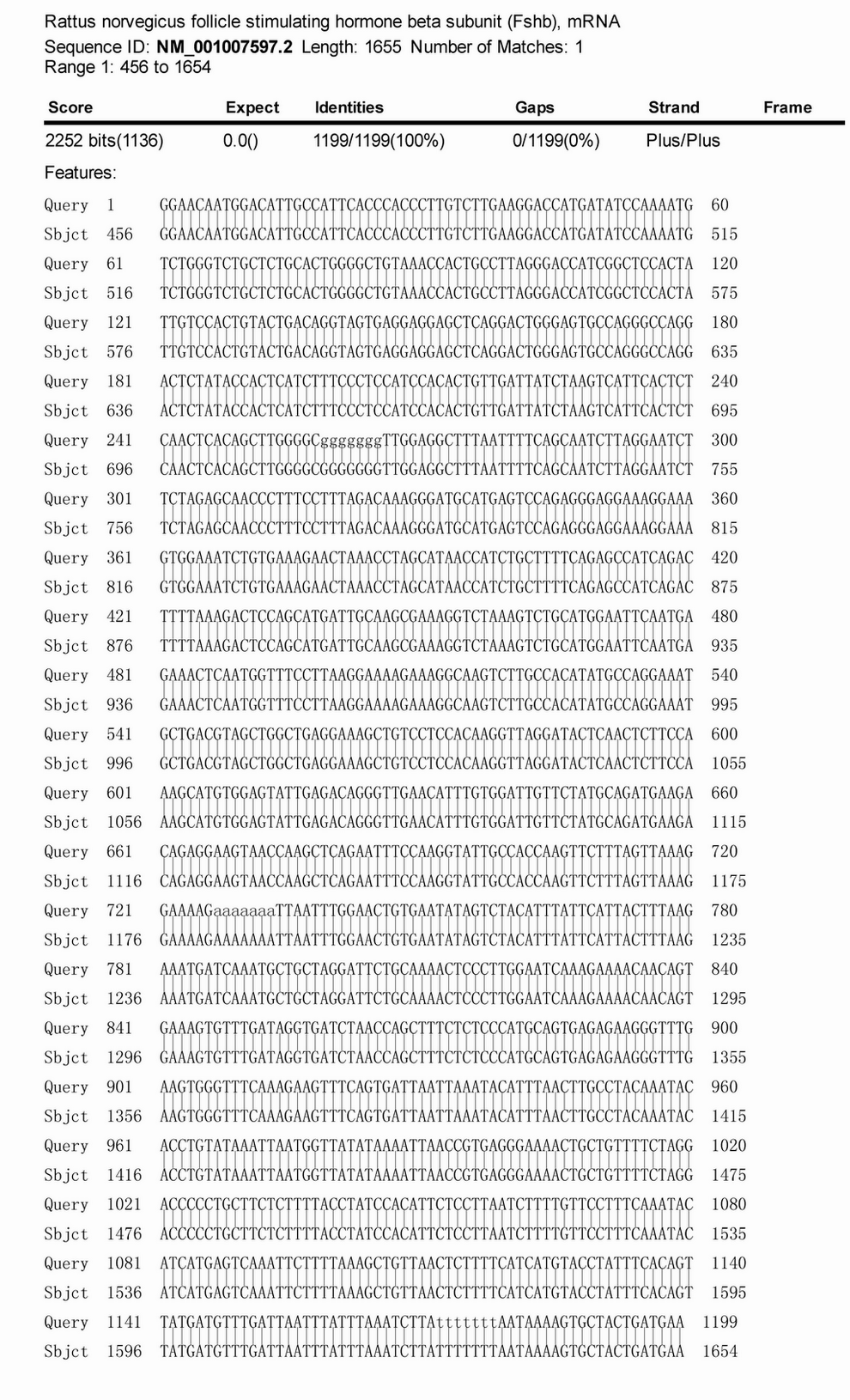


**S1. Fig. 2**. **Blast results.** Alignment of the inserted sequence of the positive clone with the sequence of the rat FSHb gene (NM_001007597.2). A total sequence of 1199 bp was identified, which showed 100% identity.
