# Supplementary material for: Roles of differential expression of microRNA-21-3p and microRNA-433 in FSH regulation in rat anterior pituitary cells
Source: Oncotarget. 2017 Mar 28;8(22):36553–65. doi: 10.18632/oncotarget.16615 (PMC5482676; doi:10.18632/oncotarget.16615)
Supplement: Supplementary file 4 [file oncotarget-08-36553-s004.docx]

**S3 File. Construction of pmiR-FSHb-3’UTR-MUT reporter plasmid**

The full-length 3’UTR of rat FSHb mRNA was cloned between the XhoI and NotI sites of the pmiR-RB-REPORT^TM^ plasmid. To disrupt the binding site of the FSHb 3’UTR, the target sequence ATCATGA (1081-1087) was mutated into TAGTACT, and TAACCAT (392-398) was mutated into ATTGGTA, forming the pmiR-FSHb-3’UTR-MUT plasmid. The primers used in the colony experiment were as follows:

FSHb F: GCGCTCGAGGGAACAATGGACATTGCC

FSHb R: AATGCGGCCGCTTCATCAGTACGACTTTA

FSHb-MUT F: GGGAAAACACGACAATTCTAGGACCCCCTGCTT

FSHb-MUT R: TCCTAGAATTGTCGTGTTTTCCCTCACGGTTAA

FSHb-MUT1 F: TCAAATACTAGTACTGTCAAATTCTTTTAAAGC

FSHb-MUT1 R: AATTTGACAGTACTAGTATTTGAAAGGAACAAA

The PCR product was analyzed via agarose gel electrophoresis (Fig. 1A). The PCR colony was identified after purification of the PCR product, enzyme cleavage, purification of the cleavage product, connection and convention. The length of the product of the PCR colony was different from that of the wild-type colony (Fig. 1B).


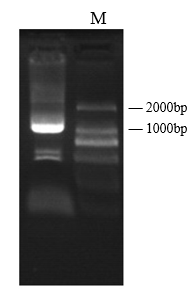

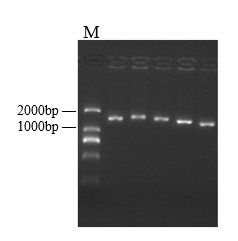


A B

**S1. Fig. 1. Results of agarose gel electrophoresis.** (A) The PCR product is compared with the markers. (B) According to the markers, the product length of the colony was different from that of the wild-type colony.

The plasmid was extracted from the colony, and the sequence was identified by a sequencing company. The results of sequencing (Fig. 2A-B) showed that the target sequences were mutated successfully.


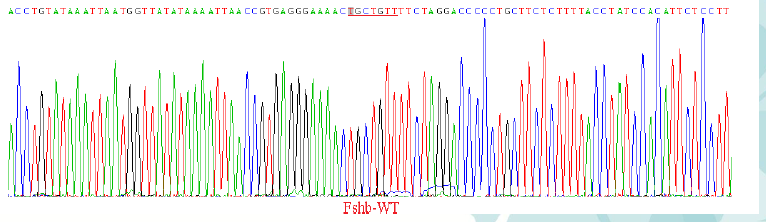

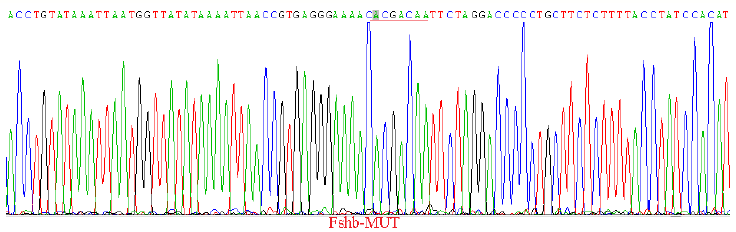


A
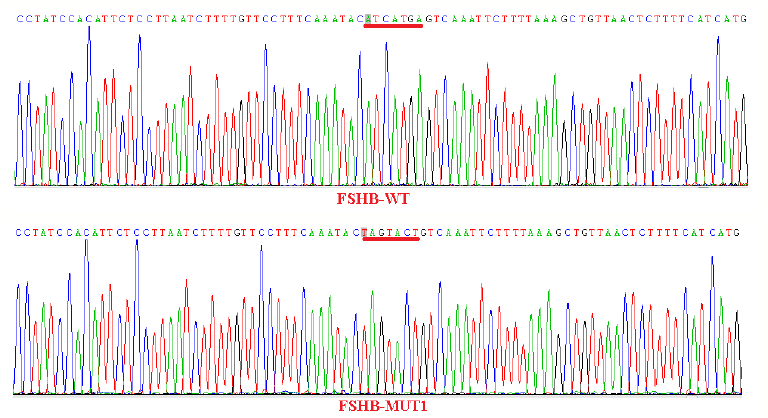

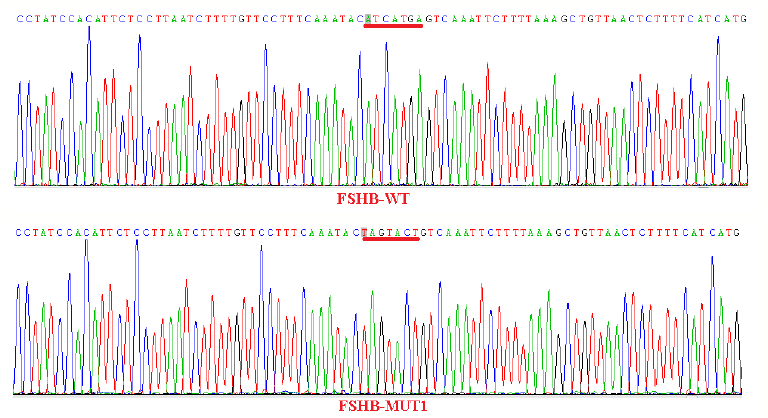


B

**S2. Fig. 2. DNA sequence peak map.** (A) Sequence of the extracted plasmid; the target sequence TGCTGTT was mutated into ACGACAA. (B) Sequence of the extracted plasmid; the target sequence ATCATGA was mutated into TAGTACT.
