# Supplementary material for: Roles of differential expression of microRNA-21-3p and microRNA-433 in FSH regulation in rat anterior pituitary cells
Source: Oncotarget. 2017 Mar 28;8(22):36553–65. doi: 10.18632/oncotarget.16615 (PMC5482676; doi:10.18632/oncotarget.16615)
Supplement: Supplementary file 5 [file oncotarget-08-36553-s005.docx]

| **miRNA** | **Position in the UTR** | **seed match** | **context++ score** | **context++ score percentile** | **weighted context++ score** | **conserved branch length** | **Pct** |
| --- | --- | --- | --- | --- | --- | --- | --- |
| rno-miR-471-3p | 1032-1038 | 7mer-1A | -0.14 | 90 | -0.14 | 0.128 | N/A |
| rno-miR-488-3p | 1032-1039 | 8mer | -0.43 | 99 | -0.43 | 0.322 | N/A |
| rno-miR-433-3p | 1044-1050 | 7mer-m8 | -0.14 | 89 | -0.14 | 0.795 | N/A |
| rno-miR-186-5p | 1056-1062 | 7mer-m8 | -0.09 | 96 | -0.09 | 0.322 | N/A |
| rno-miR-21-3p | 1067-1073 | 7mer-1A | -0.2 | 91 | -0.2 | 0 | N/A |
| rno-miR-881-3p | 1098-1105 | 8mer | -0.44 | 99 | -0.44 | 0 | N/A |
| rno-miR-505-5p | 111-117 | 7mer-1A | -0.14 | 77 | -0.14 | 0 | N/A |
| rno-miR-3548 | 1148-1154 | 7mer-1A | -0.25 | 94 | -0.25 | 0 | N/A |
| rno-miR-199a-3p | 1152-1158 | 7mer-1A | -0.18 | 86 | -0.18 | 0 | < 0.1 |
| rno-miR-6324 | 1152-1158 | 7mer-1A | -0.14 | 77 | -0.14 | 0 | N/A |
| rno-miR-344b-1-3p | 1181-1187 | 7mer-1A | -0.01 | 57 | -0.01 | 0.633 | N/A |
| rno-miR-410-3p | 1181-1187 | 7mer-1A | -0.04 | 87 | -0.04 | 0.633 | N/A |
| rno-miR-568 | 1184-1190 | 7mer-1A | -0.18 | 91 | -0.18 | 0.128 | N/A |
| rno-miR-1839-5p | 1200-1206 | 7mer-1A | -0.22 | 87 | -0.22 | 0 | N/A |
| rno-miR-196a-5p | 1200-1206 | 7mer-1A | -0.24 | 91 | -0.24 | 0 | < 0.1 |
| rno-miR-196b-5p | 1200-1206 | 7mer-1A | -0.23 | 91 | -0.23 | 0 | < 0.1 |
| rno-miR-196c-5p | 1200-1206 | 7mer-1A | -0.23 | 91 | -0.23 | 0 | < 0.1 |
| rno-miR-489-3p | 1208-1214 | 7mer-1A | -0.1 | 77 | -0.1 | 0 | < 0.1 |
| rno-miR-340-5p | 1219-1225 | 7mer-1A | -0.01 | 50 | -0.01 | 2.943 | N/A |
| rno-miR-195-3p | 1224-1230 | 7mer-m8 | -0.02 | 46 | -0.02 | 0.128 | N/A |
| rno-miR-384-3p | 1231-1238 | 8mer | -0.42 | 99 | -0.42 | 0 | N/A |
| rno-miR-216a-3p | 125-132 | 8mer | -0.43 | 99 | -0.43 | 0 | N/A |
| rno-miR-465-5p | 1318-1324 | 7mer-m8 | -0.15 | 93 | -0.15 | 0 | N/A |
| rno-miR-223-3p | 132-138 | 7mer-1A | -0.13 | 78 | -0.13 | 0 | < 0.1 |
| rno-miR-126b | 1327-1333 | 7mer-m8 | -0.16 | 93 | -0.16 | 0 | N/A |
| rno-miR-582-3p | 136-142 | 7mer-1A | -0.12 | 77 | -0.12 | 0 | N/A |
| rno-miR-323-3p | 1368-1374 | 7mer-m8 | -0.26 | 98 | -0.26 | 0.28 | N/A |
| rno-miR-6314 | 166-172 | 7mer-1A | -0.1 | 54 | -0.1 | 0 | N/A |
| rno-miR-221-5p | 168-174 | 7mer-m8 | -0.05 | 47 | -0.05 | 0.128 | N/A |
| rno-miR-328a-3p | 172-178 | 7mer-m8 | -0.13 | 73 | -0.13 | 0.506 | N/A |
| rno-miR-328b-3p | 172-178 | 7mer-m8 | -0.13 | 73 | -0.13 | 0.506 | N/A |
| rno-miR-221-5p | 175-181 | 7mer-1A | -0.12 | 72 | -0.12 | 0.556 | N/A |
| rno-miR-702-5p | 189-195 | 7mer-m8 | -0.26 | 96 | -0.26 | 0.128 | N/A |
| rno-miR-1843a-5p | 202-208 | 7mer-m8 | -0.26 | 93 | -0.26 | 0.128 | N/A |
| rno-miR-421-3p | 214-220 | 7mer-m8 | -0.22 | 94 | -0.22 | 0 | N/A |
| rno-miR-3553 | 232-238 | 7mer-m8 | -0.12 | 89 | -0.12 | 0 | N/A |
| rno-miR-1896 | 23-29 | 7mer-m8 | -0.31 | 96 | -0.31 | 0 | N/A |
| rno-miR-214-5p | 241-247 | 7mer-1A | -0.17 | 83 | -0.17 | 0 | N/A |
| rno-miR-219a-1-3p | 241-247 | 7mer-1A | -0.14 | 79 | -0.14 | 0 | N/A |
| rno-miR-742-3p | 271-277 | 7mer-1A | -0.05 | 78 | -0.05 | 0 | N/A |
| rno-miR-9a-3p | 272-278 | 7mer-1A | -0.1 | 80 | -0.1 | 0 | N/A |
| rno-miR-628 | 282-288 | 7mer-1A | -0.18 | 88 | -0.18 | 0 | N/A |
| rno-miR-3573-3p | 291-297 | 7mer-1A | -0.03 | 68 | -0.03 | 0 | N/A |
| rno-miR-145-3p | 293-299 | 7mer-m8 | -0.21 | 92 | -0.21 | 0 | N/A |
| rno-miR-296-3p | 308-314 | 7mer-m8 | -0.2 | 91 | -0.2 | 0.49 | N/A |
| rno-miR-204-5p | 326-332 | 7mer-m8 | -0.09 | 77 | -0.09 | 0 | < 0.1 |
| rno-miR-211-5p | 326-332 | 7mer-m8 | -0.09 | 77 | -0.09 | 0 | < 0.1 |
| rno-miR-188-5p | 327-333 | 7mer-m8 | -0.15 | 86 | -0.15 | 0.5 | N/A |
| rno-miR-324-5p | 329-336 | 8mer | -0.55 | 99 | -0.55 | 0 | N/A |
| rno-miR-378a-3p | 339-346 | 8mer | -0.35 | 96 | -0.35 | 0.763 | N/A |
| rno-miR-6315 | 340-346 | 7mer-1A | -0.2 | 83 | -0.2 | 0 | N/A |
| rno-miR-343 | 345-351 | 7mer-m8 | -0.25 | 92 | -0.25 | 0 | N/A |
| rno-miR-185-3p | 349-355 | 7mer-1A | -0.03 | 55 | -0.03 | 0.128 | N/A |
| rno-miR-27a-3p | 369-375 | 7mer-1A | -0.12 | 83 | -0.12 | 0.353 | < 0.1 |
| rno-miR-27b-3p | 369-375 | 7mer-1A | -0.11 | 81 | -0.11 | 0.353 | < 0.1 |
| rno-miR-673-5p | 369-375 | 7mer-1A | -0.18 | 91 | -0.18 | 0.128 | N/A |
| rno-miR-205 | 37-43 | 7mer-1A | -0.09 | 79 | -0.09 | 2.756 | 0.13 |
| rno-miR-218b | 392-398 | 7mer-m8 | -0.17 | 87 | -0.17 | 0 | N/A |
| rno-miR-760-3p | 407-414 | 8mer | -0.49 | 98 | -0.49 | 0 | N/A |
| rno-miR-5132-3p | 413-419 | 7mer-1A | -0.11 | 81 | -0.11 | 0 | N/A |
| rno-miR-206-5p | 434-440 | 7mer-1A | -0.11 | 81 | -0.11 | 0 | N/A |
| rno-miR-96-3p | 436-442 | 7mer-m8 | -0.06 | 75 | -0.06 | 0.128 | N/A |
| rno-miR-144-5p | 47-53 | 7mer-m8 | -0.02 | 18 | -0.02 | 0.128 | N/A |
| rno-miR-3589 | 490-496 | 7mer-m8 | -0.1 | 94 | -0.1 | 0 | N/A |
| rno-miR-376b-5p | 49-56 | 8mer | -0.25 | 96 | -0.25 | 0.128 | N/A |
| rno-miR-31a-5p | 516-523 | 8mer | -0.29 | 94 | -0.29 | 0 | < 0.1 |
| rno-miR-299a-3p | 521-527 | 7mer-1A | -0.08 | 66 | -0.08 | 0 | N/A |
| rno-miR-299b-3p | 521-527 | 7mer-1A | -0.08 | 66 | -0.08 | 0 | N/A |
| rno-miR-221-5p | 528-535 | 8mer | -0.43 | 98 | -0.43 | 0 | N/A |
| rno-miR-598-3p | 541-547 | 7mer-1A | -0.19 | 77 | -0.19 | 0 | N/A |
| rno-miR-185-3p | 555-561 | 7mer-1A | -0.03 | 49 | -0.03 | 0 | N/A |
| rno-miR-7a-5p | 572-578 | 7mer-1A | -0.01 | 36 | -0.01 | 0 | < 0.1 |
| rno-miR-7b | 572-578 | 7mer-1A | -0.01 | 36 | -0.01 | 0 | < 0.1 |
| rno-miR-206-5p | 579-585 | 7mer-m8 | -0.13 | 84 | -0.13 | 0 | N/A |
| rno-miR-509-5p | 586-592 | 7mer-1A | -0.13 | 81 | -0.13 | 0.556 | N/A |
| rno-miR-880-3p | 586-592 | 7mer-1A | -0.11 | 83 | -0.11 | 0.128 | N/A |
| rno-miR-339-5p | 597-603 | 7mer-m8 | -0.35 | 96 | -0.35 | 0.464 | N/A |
| rno-miR-3586-5p | 597-603 | 7mer-m8 | -0.38 | 97 | -0.38 | 0.464 | N/A |
| rno-miR-10a-5p | 598-604 | 7mer-m8 | -0.26 | 95 | -0.26 | 0.464 | < 0.1 |
| rno-miR-652-5p | 600-607 | 8mer | -0.5 | 99 | -0.5 | 0 | N/A |
| rno-miR-3593-3p | 602-608 | 7mer-m8 | -0.13 | 88 | -0.13 | 0 | N/A |
| rno-miR-3558-5p | 622-628 | 7mer-m8 | -0.09 | 69 | -0.09 | 0 | N/A |
| rno-miR-181b-5p | 624-630 | 7mer-m8 | -0.22 | 89 | -0.22 | 0 | < 0.1 |
| rno-miR-20a-3p | 626-632 | 7mer-1A | -0.14 | 85 | -0.14 | 0 | N/A |
| rno-miR-409a-5p | 639-645 | 7mer-1A | -0.19 | 81 | -0.19 | 0.128 | N/A |
| rno-miR-27b-5p | 645-651 | 7mer-1A | -0.2 | 86 | -0.2 | 0.128 | N/A |
| rno-miR-3551-3p | 652-658 | 7mer-m8 | -0.17 | 95 | -0.17 | 0 | N/A |
| rno-miR-449c-3p | 674-680 | 7mer-m8 | -0.13 | 90 | -0.13 | 0 | N/A |
| rno-miR-186-5p | 677-683 | 7mer-1A | -0.01 | 60 | -0.01 | 0.675 | N/A |
| rno-miR-128-3p | 714-720 | 7mer-1A | -0.17 | 87 | -0.17 | 0.462 | < 0.1 |
| rno-miR-27a-3p | 714-721 | 8mer | -0.41 | 98 | -0.41 | 0.128 | < 0.1 |
| rno-miR-27b-3p | 714-721 | 8mer | -0.41 | 98 | -0.41 | 0.128 | < 0.1 |
| rno-miR-673-5p | 715-721 | 7mer-1A | -0.16 | 89 | -0.16 | 0.128 | N/A |
| rno-miR-298-5p | 71-77 | 7mer-1A | -0.04 | 65 | -0.04 | 0 | N/A |
| rno-miR-148a-3p | 74-80 | 7mer-m8 | -0.16 | 77 | -0.16 | 0 | < 0.1 |
| rno-miR-148b-3p | 74-80 | 7mer-m8 | -0.16 | 77 | -0.16 | 0 | < 0.1 |
| rno-miR-152-3p | 74-80 | 7mer-m8 | -0.16 | 77 | -0.16 | 0 | < 0.1 |
| rno-miR-103-3p | 761-767 | 7mer-m8 | -0.27 | 95 | -0.27 | 0.699 | < 0.1 |
| rno-miR-107-3p | 761-767 | 7mer-m8 | -0.27 | 95 | -0.27 | 0.699 | < 0.1 |
| rno-miR-181a-5p | 762-769 | 8mer | -0.41 | 98 | -0.41 | 0.128 | < 0.1 |
| rno-miR-15b-5p | 762-769 | 8mer | -0.43 | 98 | -0.43 | 0.128 | < 0.1 |
| rno-miR-16-5p | 762-769 | 8mer | -0.41 | 98 | -0.41 | 0.128 | < 0.1 |
| rno-miR-195-5p | 762-769 | 8mer | -0.41 | 98 | -0.41 | 0.128 | < 0.1 |
| rno-miR-322-5p | 762-769 | 8mer | -0.44 | 98 | -0.44 | 0.128 | < 0.1 |
| rno-miR-497-5p | 762-769 | 8mer | -0.43 | 98 | -0.43 | 0.128 | < 0.1 |
| rno-miR-503-5p | 763-769 | 7mer-1A | -0.22 | 93 | -0.22 | 0.128 | < 0.1 |
| rno-miR-19b-1-5p | 777-783 | 7mer-m8 | -0.15 | 90 | -0.15 | 0.494 | N/A |
| rno-miR-19b-2-5p | 777-783 | 7mer-m8 | -0.16 | 91 | -0.16 | 0.494 | N/A |
| rno-miR-145-3p | 790-796 | 7mer-1A | -0.09 | 77 | -0.09 | 0 | N/A |
| rno-miR-291a-5p | 811-817 | 7mer-1A | -0.03 | 69 | -0.03 | 0 | N/A |
| rno-miR-383-5p | 820-826 | 7mer-1A | -0.11 | 75 | -0.11 | 0 | < 0.1 |
| rno-miR-133c | 827-833 | 7mer-m8 | -0.2 | 95 | -0.2 | 0 | N/A |
| rno-miR-320-3p | 829-835 | 7mer-m8 | -0.12 | 87 | -0.12 | 0.128 | N/A |
| rno-miR-185-5p | 835-841 | 7mer-m8 | -0.18 | 84 | -0.18 | 0 | N/A |
| rno-miR-30c-1-3p | 837-843 | 7mer-m8 | -0.18 | 88 | -0.18 | 0 | N/A |
| rno-miR-30c-2-3p | 837-843 | 7mer-m8 | -0.19 | 89 | -0.19 | 0 | N/A |
| rno-miR-9b-3p | 83-89 | 7mer-1A | -0.09 | 64 | -0.09 | 0 | N/A |
| rno-miR-217-5p | 843-849 | 7mer-m8 | -0.12 | 85 | -0.12 | 0.128 | < 0.1 |
| rno-miR-324-3p | 845-851 | 7mer-1A | -0.07 | 71 | -0.07 | 0.128 | N/A |
| rno-miR-320-5p | 852-858 | 7mer-m8 | -0.16 | 92 | -0.16 | 0.128 | N/A |
| rno-miR-497-3p | 858-864 | 7mer-1A | -0.11 | 79 | -0.11 | 0 | N/A |
| rno-miR-547-5p | 862-868 | 7mer-m8 | -0.1 | 74 | -0.1 | 0 | N/A |
| rno-miR-299a-5p | 86-93 | 8mer | -0.25 | 94 | -0.25 | 0 | N/A |
| rno-miR-299b-5p | 86-93 | 8mer | -0.27 | 95 | -0.27 | 0 | N/A |
| rno-miR-653-5p | 870-876 | 7mer-1A | -0.1 | 80 | -0.1 | 0.286 | N/A |
| rno-miR-181b-1-3p | 884-890 | 7mer-m8 | -0.29 | 97 | -0.29 | 0.128 | N/A |
| rno-miR-674-5p | 884-890 | 7mer-1A | -0.12 | 89 | -0.12 | 0.128 | N/A |
| rno-miR-140-5p | 89-95 | 7mer-m8 | -0.21 | 88 | -0.21 | 2.699 | 0.42 |
| rno-miR-135b-3p | 913-919 | 7mer-1A | -0.25 | 87 | -0.25 | 0.128 | N/A |
| rno-miR-223-5p | 919-925 | 7mer-m8 | -0.22 | 91 | -0.22 | 0 | N/A |
| rno-let-7f-2-3p | 926-933 | 8mer | -0.32 | 99 | -0.32 | 0.128 | N/A |
| rno-let-7a-1-3p | 927-933 | 7mer-1A | -0.11 | 88 | -0.11 | 0.556 | N/A |
| rno-let-7b-3p | 927-933 | 7mer-1A | -0.09 | 84 | -0.09 | 0.556 | N/A |
| rno-let-7c-2-3p | 927-933 | 7mer-1A | -0.11 | 88 | -0.11 | 0.556 | N/A |
| rno-let-7f-1-3p | 927-933 | 7mer-1A | -0.11 | 88 | -0.11 | 0.556 | N/A |
| rno-miR-3596d | 927-933 | 7mer-1A | -0.09 | 83 | -0.09 | 0.556 | N/A |
| rno-miR-466b-2-3p | 927-933 | 7mer-1A | -0.04 | 70 | -0.04 | 0.128 | N/A |
| rno-miR-466b-4-3p | 927-933 | 7mer-1A | -0.04 | 70 | -0.04 | 0.128 | N/A |
| rno-miR-98-3p | 927-933 | 7mer-1A | -0.09 | 83 | -0.09 | 0.556 | N/A |
| rno-miR-34a-5p | 92-98 | 7mer-m8 | -0.3 | 89 | -0.3 | 0 | < 0.1 |
| rno-miR-34b-5p | 92-98 | 7mer-m8 | -0.29 | 88 | -0.29 | 0 | < 0.1 |
| rno-miR-34c-5p | 92-98 | 7mer-m8 | -0.29 | 88 | -0.29 | 0 | < 0.1 |
| rno-miR-449a-5p | 92-98 | 7mer-m8 | -0.28 | 88 | -0.28 | 0 | < 0.1 |
| rno-miR-449c-5p | 92-98 | 7mer-m8 | -0.26 | 85 | -0.26 | 0 | < 0.1 |
| rno-miR-344b-1-3p | 941-948 | 8mer | -0.09 | 97 | -0.09 | 0.128 | N/A |
| rno-miR-410-3p | 941-948 | 8mer | -0.18 | 98 | -0.18 | 0.128 | N/A |
| rno-miR-124-3p | 95-101 | 7mer-1A | -0.01 | 41 | -0.01 | 0 | < 0.1 |
| rno-miR-760-5p | 959-965 | 7mer-1A | -0.11 | 67 | -0.11 | 0 | N/A |
| rno-miR-21-3p | 970-976 | 7mer-m8 | -0.11 | 76 | -0.11 | 0 | N/A |
| rno-miR-615 | 982-988 | 7mer-m8 | -0.26 | 87 | -0.26 | 0.128 | N/A |

**S1 Table. The 150 miRNAs predicted by TargetScan program**
